# Good response on high nasal oxygen flow reduces the need for intubation in adult respiratory failure

**DOI:** 10.1186/cc10745

**Published:** 2012-03-20

**Authors:** L Van Wagenberg, IM Hoekstra, GC Admiraal, M Slabbekoorn

**Affiliations:** 1 Medisch Centrum Haaglanden, Den Haag, the Netherlands

## Introduction

High nasal flow (HNF) therapy has proven its efficiency in acute respiratory failure when compared to conservative oxygen therapy [1]. This study was performed to find a responding and nonresponding group on HNF therapy in adults with hypoxic respiratory insufficiency measured by oxygenation and work of breathing.

## Methods

A prospective observational study during a 6-month period in patients ≥18 years with acute hypoxic respiratory failure when conservative oxygen therapy (15 l/minute) failed. Arterial blood gas analysis was done before HNF therapy and after 1 hour on flow 50 l/minute with FiO_2 _1.0. Breaths per minute and saturation were noted. When patients remained respiratory insufficient they were intubated.

## Results

A total of 20 patients was included. Mean age 63.95 ± 3 years and APACHE II score 23 ± 7. Mean PaO_2_/FiO_2 _(P/F) ratio on admission was 77.7 ± 4.2. A total of seven out of 20 patients (35%) needed endotracheal intubation. After 1 hour of HNF therapy PaO_2 _and saturation measured in arterial blood gas significantly increased from respectively 8.9 ± 0.3 kPa to 16.1 ± 2.4 kPa (*P *= 0.023) and from 91.8 ± 1.2% to 96.5 ± 0.8% (*P *= 0.001). Work of breathing, measured by the frequency of breathing, significantly decreased from 35 ± 3 times a minute to 22 ± 2 times a minute. The group that was in need of endotracheal intubation showed a less prominent response to 1-hour HNF therapy, expressed in PaO_2 _(13.2 ± 2.6 kPa vs. 16.1 ± 3.4 kPa, *P *= 0.548), saturation (94.4 ± 1.6% vs. 96.5 ± 0.8%, *P *= 0.228) and breathing frequency (25 ± 2.4/minute vs. 22 ± 2/minute, *P *= 0.357). The duration of HNF therapy was 26.1 ± 6.3 hours in the nonintubated group and 15.1 ± 9.8 hours for those who were intubated (*P *= 0.345).

## Conclusion

**Figure 1:**
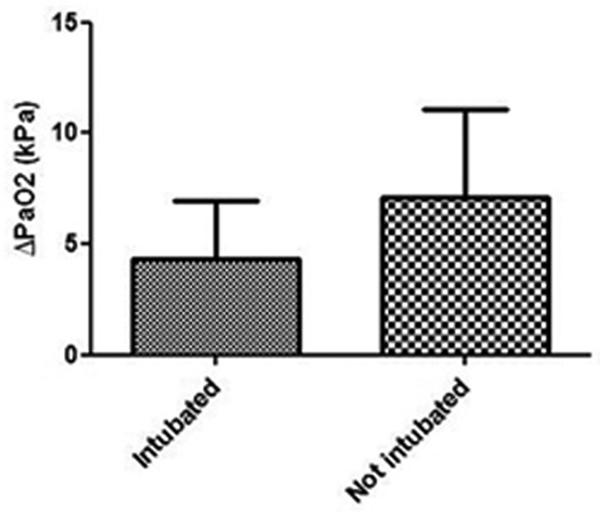
**PaO_2 _after 1-hour HNF therapy**.

All included patients did have a reduced P/F ratio and are therefore to be considered severely respiratory compromised. PaO_2 _and saturation increased with the use of HNF therapy, while work of breathing decreased. These changes were less prominent in the nonresponding group (Figure 1). The nonresponders, except one, were intubated within 15 hours after the start of HNF therapy.

## References

[B1] Roca Respir Care 2010 55 408 413 20406507

